# Does ALS‐FUS without *FUS* mutation represent ALS‐FET? Report of three cases

**DOI:** 10.1111/nan.12527

**Published:** 2018-11-20

**Authors:** S. Borrego‐Écija, E. Cortés‐Vicente, L. Cervera‐Carles, J. Clarimón, J. Gámez, J. Batlle, G. Ricken, L. Molina‐Porcel, I. Aldecoa, R. Sánchez‐Valle, R. Rojas‐García, E. Gelpi

**Affiliations:** ^1^ Neurology Department Hospital Clínic‐IDIBAPS Barcelona Spain; ^2^ Neurological Tissue Bank of the Biobanc‐Hospital Clínic‐IDIBAPS Barcelona Spain; ^3^ Neurology Department Institut d'Investigacions Biomèdiques‐Hospital de la Santa Creu i Sant Pau Universitat Autònoma de Barcelona Barcelona Spain; ^4^ Center for Networked Biomedical Research into Neurodegenerative Diseases (CIBERNED) Madrid Spain; ^5^ Neurology Department Hospital de la Vall d'Hebron Barcelona Spain; ^6^ Neurology Department Hospital Sant Pau y Santa Tecla Tarragona Spain; ^7^ Institute of Neurology Medical University of Vienna Vienna Austria; ^8^ Pathology Department CDB, Hospital Clinic Barcelona Barcelona Spain

Abnormal cytoplasmic accumulation of fused in sarcoma (FUS) protein is the pathological hallmark of some cases of amyotrophic lateral sclerosis (ALS) with transactive response DNA‐binding protein of 43KDa (TDP‐43)‐negative pathology that lack SOD1 mutations. FUS is an RNA‐binding protein located predominantly in the nucleus and is involved in regulation of transcription, alternative splicing, RNA stability, microRNA biogenesis, apoptosis and cell division. FUS, Ewing's sarcoma (EWS) and TATA‐binding protein‐associated factor 15 (TAF15) proteins constitute the FET (FUS/EWS/TAF15) family, highly conserved and ubiquitously expressed RNA‐binding proteins that shuttle between nucleus and cytoplasm assisted by the nuclear import protein Transportin 1 (Trn1) 1.

Accumulation of FUS also occurs in other related neurodegenerative conditions such as atypical frontotemporal lobar degeneration with ubiquitinated inclusions (aFTLD), neuronal intermediate filament inclusion disease (NIFID) and basophilic inclusion body disease (BIBD), the three currently recognized forms of frontotemporal lobar degeneration with FUS pathology (FTLD‐FUS) 2.

Recent work suggests different pathological processes underlie ALS‐FUS and FTLD‐FUS. First, most ALS‐FUS cases are caused by *FUS* mutations 3, while most FTLD‐FUS cases are not 2, 4. Neumann *et al*. described that in ALS‐FUS, the cytoplasmic inclusions consist solely of FUS protein while in FTLD‐FUS, the inclusions include other FET family proteins such as TAF15 or EWS 5. In addition, they observed that Trn1, a protein involved in the nuclear transport, accumulates specifically in FTLD‐FUS inclusions but not in ALS‐FUS. These findings led the authors to suggest that ALS with *FUS* mutations is more restricted to FUS dysfunction, while in FTLD‐FUS, there is a more global and complex dysregulation of all FET proteins. They suggest changing the nomenclature and recommended using the term FTLD‐FET for FTLD‐FUS but to preserve the term ALS‐FUS 5.

We describe three cases of ALS‐FUS with TAF15 and Trn1 accumulation in which *FUS* mutations were not detected. Brain donors and/or next of kin had given their written informed consent for the use of brain tissue for research, and the research protocol has been approved by the Ethics Committee of the Hospital Clinic Barcelona.

Patient 1, a 63‐year‐old man, developed slowly progressive weakness in the distal muscles, dysarthria and dysphagia. Neurological examination revealed symmetrical weakness and hyperreflexia, fulfilling the criteria for ALS. Cognitive and behavioural symptoms were not reported during follow‐up. He died of respiratory failure at 69 years. After brain donation, the unfixed brain weight was 1390 g. A prominent atrophy of the medullary pyramids, anterior nerve roots and spinal cord was appreciated on gross examination, but without brain atrophy. Histologically, prominent neuronal loss of motor neurones of the anterior horn was observed at all levels of the spinal cord and was also present in the motor nuclei of the brain stem and the primary motor cortex. Degeneration of the corticospinal tracts was also observed. Several of the remaining spinal and cortical motor neurones showed relatively large cytoplasmic basophilic inclusions. These inclusions were also observed in nonmotor pyramidal neurones and were partly basophilic and partly fibrillar. These inclusions were immunoreactive for FUS protein, p62, TAF15 and Trn1 (Figure 1 **A**1–**A**5), and partially for ubiquitin, alpha‐internexin and phosphorylated neurofilaments. These findings were consistent with ALS with FUS‐positive basophilic and fibrillary inclusions.

**Figure 1:**
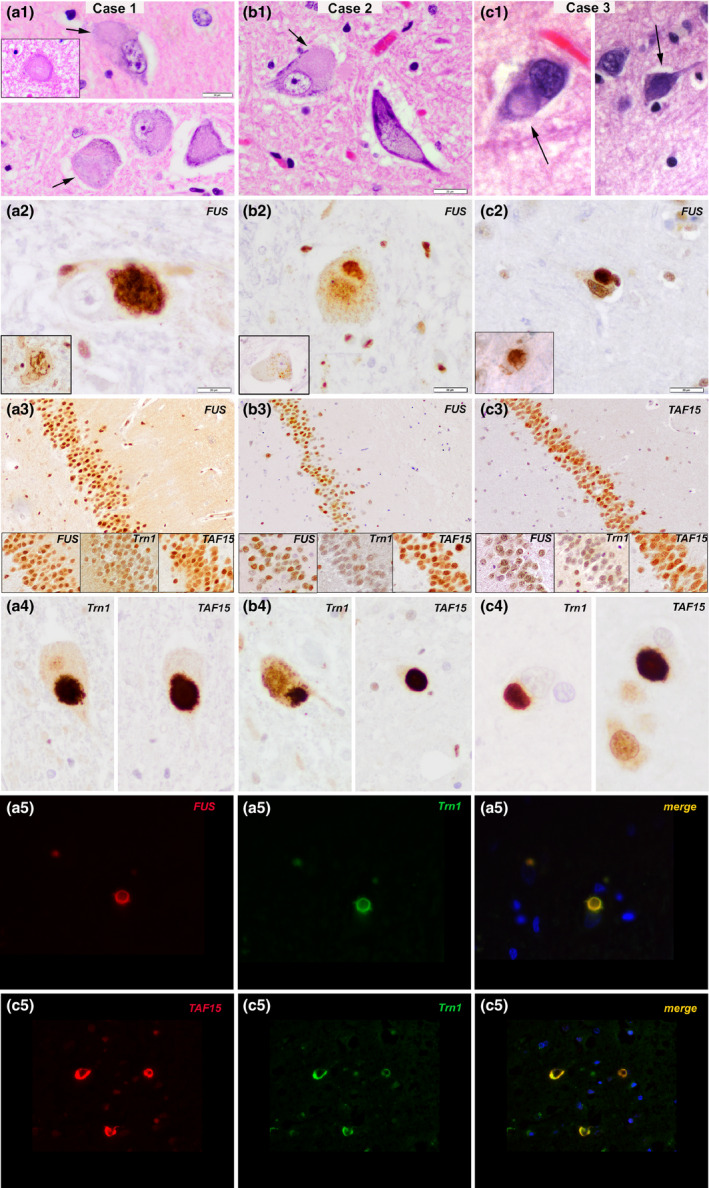
Representative neuropathological findings in the three cases: (**A1**,** B1**,** C1**) HE‐stained sections show different types of intraneuronal inclusion bodies in the motor neurones of the frontal cortex, brainstem and spinal cord (arrows) varying in shape and tinctorial properties (basophilic, pale, with a condensed centre or with fibrillar appearance). (**A2, B2, C2**) Inclusions are FUS‐positive and appear either compact, more fibrillar or skein‐like (inset) (immunohistochemistry for FUS; slightly counterstained with haematoxylin). (**A3, B3, C3**) There is no involvement of the dentate gyrus of the hippocampus, and granule cells are devoid of FUS/TAF15/Trn1 +  inclusion bodies (immunohistochemistry for FUS (**A**3, **B**3 and insets), TAF15 (**C**3 and insets) and Transportin 1 (Trn1)(insets)). (**A4, B4, C4**) Intraneuronal inclusion bodies in motor cortex, brainstem and spinal cord neurones are also strongly immunoreactive for Transportin 1 and TAF15 (immunohistochemistry for Transportin 1 (Trn1) and TAF15 shown in the left and right panel, respectively; slightly counterstained with haematoxylin). **A**5: Double immunofluorescence for FUS (red, left panel), Trn1 (green, middle panel) and merged image (yellow‐orange, right panel) shows codistribution of both proteins in the same inclusion body in patient 1. **C**5: Double immunofluorescence for TAF15 (red, left panel), Trn1 (green, middle panel) and merged image (yellow‐orange, right panel) shows codistribution of both proteins in the same inclusion body in patient 3. **A**1–**A**5 are from patient 1, **B**1–**B**4 are from patient 2, and **C**1–**C**5 are from patient 3. *Scale bars: A1, B1, C1, A2, B2, C2, A4, B4, C4: 20 μm, A3, B3, C3: 50 μm*.

Patient 2, a 71‐year‐old woman, presented with progressive weakness of lower extremities, dysarthria and dysphagia. Neurological examination revealed pyramidal signs. No lower motor neurone signs were found on examination, and she was diagnosed with primary lateral sclerosis. During the disease course, she developed an akinetic–rigid syndrome without response to levodopa. DAT‐SPECT showed bilaterally reduced putaminal tracer uptake. No cognitive symptoms were reported. The patient died at the age of 83 years after a total disease duration of 12 years. After brain donation, the unfixed brain weight was 1035 g. Gross examination showed moderate brain atrophy with preferential involvement of the frontotemporal regions. Diffuse nigral pallor was also observed. Histologically, severe loss of motor neurones at all levels of the spinal cord and brain stem nuclei was observed. In contrast, no prominent neuronal loss of primary motor cortex neurones and no unequivocal signs of corticospinal tract degeneration were identified. In addition, there was a depletion of pigmented neurones of the substantia nigra and neuronal loss and gliosis of the subthalamic nucleus and internal pallidum. Residual motor neurones of the spinal cord and hypoglossal nucleus showed relatively large, faintly basophilic inclusions that showed strong FUS immunoreactivity (Figure 1 **B**1–**B**4) and were negative for ubiquitin, neurofilaments and TDP‐43. Some FUS‐positive glial inclusions were also identified. Most of these inclusions showed immunoreactivity for TAF15 and Trn1 antibodies (Figure 1 **B**4). The final diagnosis was motor neurone disease with preferential involvement of lower motor neurones with pallidoluysian atrophy and nigral degeneration with abundant neuronal and lesser glial FUS‐positive inclusions. Concomitantly, advanced Alzheimer's disease neuropathological change (A3, B3, C3 score according to the NIAA/AA consensus criteria) was found 6.

Patient 3, a 43‐year‐old man, presented with leg weakness. On neurological examination, there was generalized amyotrophy, fasciculations and hyperreflexia. He developed dysarthria and dysphagia during follow‐up and died of pneumonia at the age of 48 years. Cognitive and behavioural symptoms were not reported. The clinical diagnosis was ALS. He had no family history of ALS or dementia. After brain donation, the unfixed brain weight was 1500 g. Gross examination revealed mild brain atrophy with preferential involvement of the precentral and postcentral gyri. Histologically, loss of motor neurones was evident in the primary motor cortex, hypoglossal nuclei and also at all levels of the spinal cord. Moreover, in the pre‐ and postcentral regions as well as in the temporal cortex, laminar spongiosis and gliosis were evident in superficial cortical layers. While with H&E staining, inclusions were difficult to identify (Figure 1 **C**1), immunohistochemistry for FUS showed frequent neuronal cytoplasmic inclusions (Figure 1 **C**2), short neurites and few intranuclear inclusions. Inclusions were more abundant in the precentral gyrus, in the brainstem nuclei and in the spinal cord. They were also immunoreactive for TAF15 and Trn1 (Figure 1 **C**4–**C**5) and were negative for TDP‐43. The final diagnosis was ALS‐FUS. In all three cases, granular neurones of the dentate gyrus were devoid of inclusions (Figure 1 **A**3, **B**3, **C**3 and insets).

Genetic analysis of the *FUS* gene was performed in the three donors. All 15 *FUS* exons including intron–exon flanking regions, as well as the 3′UTR region of *FUS* gene, were amplified through PCR. Final PCR products were purified and Sanger‐sequenced using BigDye terminator chemistry (Applied Biosystems). Sequences were run on an Applied Biosystems^®^ 3130 Genetic Analyzer, and resulting electropherograms were visually inspected using Sequencher (version 4.1, Gene Codes Corp.). Genetic analysis did not disclose any *FUS* mutation in any of these three patients. Since several variants in the 3′ untranslated region (3′UTR) of the *FUS* gene have been described with uncertain pathogenicity (that is c.∗48G>A, c.∗59G>A, c.∗108C>T and c.∗110G>A) 7, 8, we also screened the genomic region containing these variants. We only found one patient harbouring the c.*41G>A rare heterozygous variant (rs80301724) 9. Previous studies have reported this polymorphic variant to be equally present in ALS cases and controls, thus showing a lack of genetic association between this particular nucleotide change and ALS 8, 10.

Here, we describe the clinicopathological phenotype of three ALS patients with abundant FUS‐positive protein aggregates. The inclusion bodies were also immunoreactive for TAF15 and Trn1, and no mutation in the *FUS* gene was detected. Similar cases had been reported in Japan by Matsuoka *et al*., Fujita *et al*. and Takeuchi *et al*. (Table 1)^11^, ^12^, ^13^. Other possible genes that could have mutations include *TPN1* and *TAF15*, among others, that were not tested in our cases.

**Table 1:** Demographic and clinical features of ALS‐FUS cases in the literature

	Present study			Fujita *et al*. 10	Matsuoka *et al*. 11	Takeuchi *et al*. 12
	Patient 1	Patient 2	Patient 3			
Gender	Male	Female	Male	Female	Female	Female
Family history	No	No	No	No	No	No
FUS mutation	No	No	No	No	No	No
Age at onset (y)	63	71	43	73	75	73
Age at death (y)	69	83	48	75	79	75
Motor neurone	Yes	Yes	Yes	Yes	Yes	Yes
Onset	Spinal	Spinal	Spinal	Spinal	Spinal	Spinal
Dementia	No	No	No	No	No	No
Parkinsonism	No	Yes	No	No	No	No
Neuropathology	ALS‐FUS	ALS‐FUS	ALS‐FUS	ALS‐FUS	ALS‐FUS	ALS‐FUS
FUS IHC	+	+	+	+	+	+
TAF15 IHC	+	+	+	NE	NE	+
TRN1 IHC	+	+	+	NE	NE	+

ALS, amyotrophic lateral sclerosis; FUS, fused in sarcoma; NE, not evaluated; y, years; IHC, immunohistochemistry.

These findings differ from the ALS‐FUS cases previously reported by Neumann and behave immunohistochemically similar to FTLD‐FET cases. Whether these cases might be specific to certain populations is unresolved. Based on our results, we confirm the concept that the presence of FET and Trn1 proteins within the inclusions is strong indicator of a lack of pathogenic mutations within *FUS*. However, this immunohistochemical profile does not differentiate between an ALS and FTLD phenotype. If we hypothesize that FTLD‐FUS with *FUS* mutations will not show Trn1 or any other FET family protein than FUS, a change of the nomenclature in the ALS‐FUS and FTLD‐FUS with no mutations of *FUS* should be considered, and the use of the terms ALS‐FET and FTLD‐FET might be more appropriate.

ALS‐FUS mutation cases seem to have different morphological phenotypes depending on the age of onset or disease duration; neuronal basophilic inclusions being more frequently detected in early juvenile forms, while fibrillary or tangle‐like inclusions and glial inclusions tend to appear in late‐onset cases 3. Similar findings have been described in some sporadic FTLD‐FUS cases 4, 14. Interestingly, ALS‐FUS cases without *FUS* mutations seem to have an older age of onset and a less aggressive progression than cases with mutations 3.

While some reports have detected *FUS* mutations in ‘juvenile ALS with basophilic inclusions’ 15, others have not found mutations in the adult‐onset group 11, 12. It might be therefore that a subgroup of ‘adult‐onset ALS with basophilic inclusions’ represents the ALS counterpart of basophilic inclusion body disease and may therefore be considered an ALS‐FET subtype without *FUS* mutations.

Our study expands the clinicopathological spectrum of nongenetic ALS‐FUS cases and reinforces the idea that not all ALS‐FUS cases are secondary to *FUS* mutations. It also corroborates the usefulness of TAF‐15 and Trn1 immunohistochemistry for the neuropathological diagnosis of nongenetic FTLD‐FET and ALS‐FET patients. Whether these cases represent a different pathogenetic subgroup among ALS‐FUS is unclear and requires further detailed clinical, neuropathological and molecular studies.

## Authors’ Contributions

Study concept and design: SBE, RRG, EG; Acquisition of data: SBE, ECV, RRG, LCC, GR, JG, JB, EG; Analysis and interpretation of data: SB, JC, EG; Drafting of the manuscript: SB, EG; Critical revision of the manuscript and editing: All authors.

## Conflict of Interest

The authors do not report conflict of interests related to this article.
